# Enhanced Risk Prediction for Coronary Heart Disease by Leveraging Polygenic Risk Score and Clinical Risk Score in European Hypertensive Adults

**DOI:** 10.3390/jcdd12120454

**Published:** 2025-11-24

**Authors:** Siyuan Shen, Yuquan Wang, Yue-Qing Hu

**Affiliations:** State Key Laboratory of Genetics and Development of Complex Phenotypes, Institute of Biostatistics, School of Life Sciences, Fudan University, Shanghai 200438, China; lenop2ssy@163.com (S.S.); 21110700112@m.fudan.edu.cn (Y.W.)

**Keywords:** coronary heart disease, hypertension, polygenic risk score, risk factors

## Abstract

Background: Coronary heart disease (CHD) is the leading cause of premature mortality. The incremental value of a polygenic risk score (PRS) to a clinical risk score towards improving CHD prediction is controversial. Meanwhile, the effect of PRSs on CHD prediction in the chronic disease population is unclear. Methods: Utilizing publicly available summary statistical data, we developed several PRSs using the genome data of European ancestry from the Atherosclerosis Risk in Communities Study. Furthermore, we investigated the association of CHD with the best-performing PRS in both the overall and chronic disease cohorts. Additionally, we evaluated whether adding the best-performing PRS to the clinical risk score improves risk prediction. Results: A total of 6152 subjects (767 CHD cases) were included in this study. The high values from the developed best-performing PRS were significantly associated with an increased risk of CHD, with a stronger association in the hypertensive population (interaction *p* = 0.0144). Compared with individuals in the bottom 20% of the PRS values, those in the top 20% were more than 3-fold more likely to develop CHD in the overall cohort, rising to 5-fold in the hypertensive cohort. Adding PRS to the clinical risk score significantly improved the C-index (0.72 to 0.74; *p* = 0.004), with a 10% net reclassification improvement overall. The hypertensive population showed the greatest improvements. Furthermore, we observed a significant gradient of 10-year and lifetime risk of CHD based on the PRS within each clinical risk category. Conclusions: Compared to the clinical risk score, integrating the PRS significantly improved CHD prediction and better identified CHD risk trajectories, especially in the European hypertensive adult population.

## 1. Introduction

Coronary heart disease (CHD) is a complex condition arising from a combination of genetic, cardiometabolic, behavioral, environmental, and social risk factors. It remains the leading cause of premature mortality in both developed and developing countries [1,2]. Reducing the burden of CHD worldwide and providing accurate risk prediction of incident CHD is a crucial public health problem [3].

Although the treatment methods for CHD are rapidly increasing, identifying and selecting the patients who can benefit from these treatments remains a challenge. Currently, several clinical models have been proposed for predicting an individual’s incident CHD, such as the American College of Cardiology/American Heart Association Pooled Cohort Equation (PCE) [4], the European Society of Cardiology SCORE2 model [5], and the QRISK3 model [6]. These models integrate data on demographics and cardiovascular risk factors to estimate the 10-year risk of incident CHD. However, conventional clinical risk prediction models are not highly accurate in estimating the risk of CHD. For example, many individuals with an estimated 10-year CHD risk of below 7.5% based on PCE have experienced incident CHD [7]. Therefore, there is particular interest in proposing tools to further enhance the risk stratification of the population, thereby minimizing treatment costs, improving patient prognosis, and informing public health.

The estimated heritability of CHD ranges between 40% and 60%, with the majority of the heritable risk attributed to a polygenic component [8]. Polygenic risk scores (PRSs), which can capture a proportion of heritability [9], have proven to be an effective tool for predicting various diseases [10,11,12,13]. The score is the weighted sum of identified single-nucleotide polymorphisms (SNPs) associated with the interested traits or diseases in genome-wide association studies (GWASs) [14]. The successful application of PRSs to complex diseases demonstrates their strong potential for enhancing CHD risk prediction by leveraging information from millions of genomic variants. Recently, many PRSs for CHD have been developed to improve risk prediction. Several studies have shown that PRSs can improve risk prediction accuracy for prevalent CHD events compared with conventional clinical risk factors [15,16] and enhance power in terms of net reclassification improvement (NRI) by combining clinical risk prediction models (i.e., PCE) with PRSs [17,18]. In contrast, several studies [19,20] that integrated a PRS into the PCE found that the combined strategy failed to significantly improve discrimination, calibration, or risk reclassification. However, the tool’s applicability in individuals with prior chronic conditions (such as hypertension, diabetes, or chronic kidney disease) has not been fully studied. These populations are often at elevated baseline risk for CHD, but their risk trajectories differ because of complex interactions between clinical and genetic factors [21].

To address the unmet need of individualized CHD risk prediction, we constructed a CHD PRS and aimed to (1) evaluate its predictive power across subgroup populations defined by chronic disease status; and (2) assess whether incorporating CHD PRS into an established clinical arteriosclerotic cardiovascular disease (ASCVD) risk score improves CHD risk prediction. We found that the developed PRS exhibited a significant interaction in subgroups defined by chronic disease status and demonstrated excellent predictive performance in the hypertensive population. Furthermore, integrating this PRS with a clinical risk score significantly enhanced CHD risk prediction.

## 2. Materials and Methods

### 2.1. Study Population

**Figure 1:**
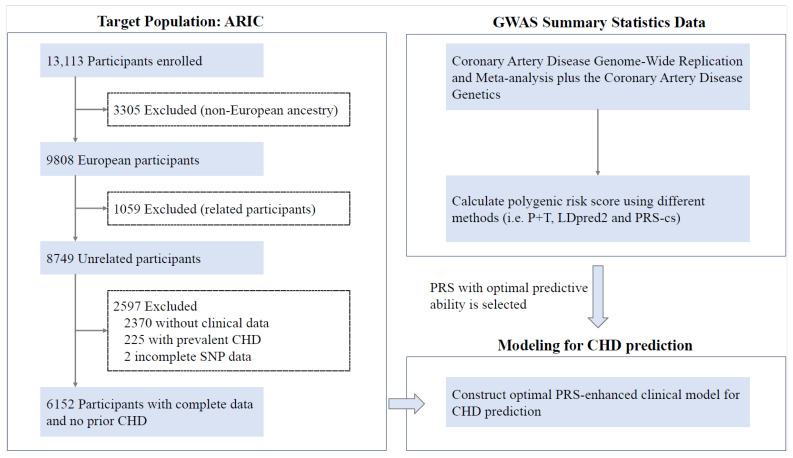
The design and flowchart of this study. To select an optimal PRS for CHD prediction, we evaluated the performance of five PRSs derived from summary statistics from the CARDIoGRAMplusC4D and an existing PRS obtained from Khera et al. Lastly, we evaluated the performance of a clinical risk model (i.e., PCE) in identifying risk of CHD and a clinical risk model combined with the PRS (i.e., the PRS-enhanced clinical risk model). CARDIoGRAMplusC4D, the Coronary Artery Disease Genome-Wide Replication and Meta-analysis (CARDIoGRAM) plus the Coronary Artery Disease (C4D) Genetics; GWAS, genome-wide association analysis; CHD, coronary heart disease; PRS, polygenic risk score.

The study population of interest included 13,113 participants from the Atherosclerosis Risk in Communities (ARIC) Study. The ARIC study is a prospective, longitudinal cohort of middle-aged black and white participants (45% male vs. 55% female) recruited from four communities in the United States (Forsyth County, North Carolina; Jackson, Mississippi; suburbs of Minneapolis, Minnesota; and Washington County, Maryland) from 1987 to 1989 (visit 1). All participants involved had a follow-up every three years until visit 4 (1996–1998), and more recently, visit 5 (2011–2013), visit 6 (2014–2016), and visit 7 (2017–2019) [22]. Clinical examination, blood measurement, physician assistant review, and a telephone questionnaire were carried out at each follow-up. This study conducted a series of screening steps to excluded participants: Step (1) excluded 3305 non-European ancestry participants; Step (2) excluded 1059 related participants; and Step (3) excluded 2370 participants without clinical data, 225 participants with prevalent CHD, and 2 participants with incomplete genotype data (Figure 1). In the end, a total of 6152 participants were included in this study. The screened dataset was randomly split into a training set (70%) and a validation set (30%). The training set was used to construct predictive models, and the validation set was used for parameter tuning of PRS algorithms. The ARIC study protocol was approved by the Institutional Review Board of Vanderbilt University Medical Center, and all participants provided written informed consent. The publicly available ARIC data can be obtained from dbGaP (phs000280).

### 2.2. Genotype Data

SNP genotype data of all participants were acquired from the Affymetrix 6.0 DNA microarray platform (Affymetrix, Santa Clara, CA, USA) and analyzed using the Birdseed variant-calling algorithm. Quality control was conducted using PLINK 2.0 [23] before genotype imputation. We excluded SNPs that had a call rate < 95%, missing data > 5%, Hardy–Weinberg equilibrium *p* values < 10^−6^, or minor allele frequencies < 5%, as well as duplicated SNPs [24]. Individuals who failed the X-chromosome sex concordance check, as well as those with more than 5% missing data, were removed. Related individuals were excluded by randomly removing one of each pair of individuals with genetic relatedness of more than 0.8. The 1000 Genomes Project was used as a reference panel for genotype imputation via an algorithm on the Michigan Server [25,26]. SNPs with an imputation quality of below 0.4 were removed. The QC procedure ensured that there was no bias produced by the sample batch effect or genotyping quality. The SNPs included by genotyping were subsequently used to calculate the PRS.

### 2.3. ASCVD Risk Score

The ASCVD 10-year risk score of all participants with available baseline data was calculated using the PCE model [4], which incorporates variables of sex, age, race and ethnicity, diabetes, total cholesterol, high-density lipoprotein cholesterol (HDL-C), systolic blood pressure (SBP), antihypertensive medication use, and smoking status. All the available baseline variables can be extracted from the phenotype dataset in the ARIC study. Specifically, the ASCVD 10-year risk score was calculated by weighting the above variables, and the corresponding weight of each variable can be obtained from reference [4]. Each participant was designated to one of three risk categories: low (<7.5%), intermediate (7.5–20%), and high (>20%), based on a 10-year risk score of CHD.

### 2.4. PRS

The CHD PRS of an individual was calculated as the weighted sum of risk alleles using the GWAS summary data from the Coronary Artery Disease Genome-Wide Replication and Meta-analysis (CARDIoGRAM) plus the Coronary Artery Disease (C4D) Genetics (CARDIoGRAMplusC4D) Consortium [27]. We computed four PRSs; one was previously developed by Khera et al. [8], and three others were developed using different algorithms (clumping and thresholding [28] (C + T/P + T), LDpred2 [29] and PRS-cs [30]). For P + T, we created 30 candidate P + T PRSs on the basis of 10 *p* value thresholds and three SNP linkage disequilibrium correlations. For LDpred, we created 21 LDpred PRSs corresponding to seven different fractions of the causal variants of three heritabilities. For PRS-cs, we created three PRS-cs PRSs corresponding to three global shrinkage parameters. Detailed information on each PRS algorithm and its tuning parameters is described in the Supplementary Materials. The optimal parameter of each algorithm was selected via parameter tuning on a separate dataset.

For the PRS tuning, the candidate PRS-cs PRSs, LDpred2 PRSs, and P + T PRSs were calculated in a validation dataset in the ARIC independent from the training dataset. The best PRS of each approach was selected using the area under the receiver operating characteristic (ROC) curve (AUC). Specifically, we fitted a logistic regression with CHD as the outcome; each candidate PRS as exposure; and age, sex, and the first ten principal components (PCs) of genotype as covariates. The best PRS-cs PRS, best LDpred2 PRS and best P + T PRS were used to select a best-performing PRS for subsequent analysis. We calculated a series of metrics to assess the performance of the best P + T PRS, best LDpred2 PRS, and best PRS-cs PRS using the same approach, with adjustment of the same set of covariates as in the tuning step through 10-fold cross validation in the whole dataset. Each PRS was standardized using z-score transformation. Based on the most predictive PRS, the study participants were divided into three genetic risk categories: low (bottom quintile), intermediate (the second to fourth quintile), and high (top quintile), as in the published approach [31].

### 2.5. Outcome and Follow-Up

The incident CHD cases were defined as having incident myocardial infarction (MI), heart attack, fatal coronary event, or silent MI detected by electrocardiogram; or having undergone a revascularization procedure by 31 December 2004. The interested variables age, smoking status (current, former and never), SBP, hypertension, antihypertensive medication use, HDL-C, stroke, congestive heart failure (CHF), chronic kidney disease (CKD), obesity, dyslipidemia, and diabetes were ascertained at the visit 1 examination. The follow-up time for each participant was calculated as the interval between the date of the baseline examination and the date of occurrence of CHD, the date of death, or the last follow-up visit, whichever occurred first.

### 2.6. Statistical Analysis

Baseline characteristics of overall participants were described as means for continuous variables and frequencies for categorical variables. To compare the differences in demographics and clinical factors for those with and without incident CHD, a two-sided Student’s t-test was used for continuous variables and a chi-squared test was used for categorical variables. To measure the overall prediction accuracy of PRSs, we calculated (i) the proportion of variation in CHD status explained by the PRS on the liability scale; (ii) the AUC for the PRS combined with covariates; and (iii) the odds ratio (OR), which measures the association of the PRS with CHD.

Two Cox proportional hazard models were constructed to investigate the association between the PRS and incident CHD and estimate the hazard ratio (HR) for the risk of incident CHD given one-unit standard deviation (SD) increment in the PRS and by quintiles of the PRS distribution. Model 1 included age, sex, and the first ten PCs; model 2 additionally included diabetes, LDL-C, HDL-C, SBP, DBP, hypertension, antihypertension, and smoking status. A series of models incorporating interaction terms between PRSs and chronic disease status were constructed to assess potential effect modification on incident CHD risk, evaluated on a continuous scale. The Cox proportional hazard model fit was estimated by examining Schoenfeld residuals to evaluate the proportional hazards assumptions for the covariates, Martingale residuals to assess nonlinearity, and deviance results to identify influential outliers. Kaplan–Meier survival analyses were conducted to explore the relationship between genetic risk categories and the cumulative incidence of CHD, with statistical comparisons performed using the log-rank test.

The Harrell concordance index (C-index) was calculated for the Cox proportional hazard models adjusted for age, sex, and the first ten PCs to examine the models’ goodness-of-fit. The incremental value of the PRS to the clinical ASCVD risk score was assessed by evaluating the difference in C-index. We calculated 95% confidence intervals for the C-index and for the difference in C-index values between models by 10-fold cross-validation using a nonoverlapping 9:1 split. We evaluated the calibration of risk prediction models by comparing the observed with the predicted event probabilities using the Greenwood–Nam–D’Agostino chi-squared test [32]. The NRI was calculated for the combined model (includes clinical ASCVD risk score and PRS, denoted by PRS-enhanced model) and the PCE model (includes clinical ASCVD risk score only). We calculated both categorized NRI and continuous NRI to assess the PRS prediction performance when added to the clinical ASCVD risk score. The 95% confidence intervals for NRI were obtained using bootstrapping. All statistical tests were two-sided and a *p*  <  0.05 was considered significant. Statistical analysis was performed in R software, version 4.3.1.

## 3. Results

### 3.1. Baseline Characteristics of the Study Cohort

A total of 6152 participants were included in our analysis (Figure 1), and 767 of them experienced a CHD event. The mean age was 54 years (SD, 5.6 years), and 44.73% of the overall cohort were male. As shown in Table S1, most of the baseline characteristics significantly differed between those who developed CHD and those who did not develop CHD. Those with CHD were older than those without CHD, with a mean age of 55.4 years (SD, 5.4 years) compared to 53.8 years (SD, 5.6 years) (*p* = 8.21 × 10^−13^). There were more men (70.3% male vs. 29.7% female, *p* = 3.26 × 10^−52^) and more former or current smokers (26.99% current smoking vs. 42.76% former smoking vs. 30.25% never smoking, respectively, *p* = 1.24 × 10^−15^) among those who developed CHD. Furthermore, SBP was significantly higher in those with CHD than those without CHD (*p* = 2.58 × 10^−19^), and individuals with CHD were also more likely to be on antihypertensives. In the context of chronic diseases, individuals with CHD had a higher incidence of hypertension, obesity, dyslipidemia, and CHF (*p* < 0.001), but not stroke or CKD. There was also a significant difference across genetic risk categories (Table S2). The mean follow-up time for those with CHD was lower than for those without CHD (10.14 years vs. 16.23 years, *p* < 0.001).

### 3.2. Performance of CHD PRS

Across the entire study population, the optimal-performing PRS with an AUC of 0.72 (95%CI: 0.68–0.76), liability R^2^ of 6.30%, and odds ratio (OR) of 1.65 (95%CI: 1.52–1.79) was calculated using the LDpred2 algorithm (Table S3). The distribution of the optimal-performing PRS in the ARIC population-based cohort shows that individuals with CHD have higher PRS values than those without CHD (Figure S1A). As shown in Figure S1B, the prevalence of CHD increases as the PRS percentile increases. In the group with the high PRS values, the prevalence of CHD was 34.78%, which is a 10.4-fold difference compared with a CHD prevalence of 3.33% in the low PRS group. Having illustrated the association between PRSs and CHD, we evaluated effect sizes in the general population. The individuals with PRS values in the highest centile (prevalence = 34.78 cases per 1000 individuals) had a significantly higher risk than those with values in the median centile (prevalence = 16.37 cases per 1000 individuals, OR = 2.53, *p* = 0.0043) and lowest centile (prevalence = 3.33 cases per 1000 individuals, OR = 23.29, *p* = 0.0023, Figure S1B). The association of CHD PRSs and CHD risk was investigated, and the CHD PRS was found to be significantly associated with CHD risk (hazard ratio (HR) per 1-SD increment = 1.58, 95%CI: 1.47–1.70, *p* = 4.73 × 10^−35^, Table 1). After adjusting for diabetes, LDL-C, HDL-C, SBP, DBP, hypertension, antihypertension, and smoking status, there was a significant association of CHD PRSs with CHD risk (HR = 1.51, 95%CI: 1.40–1.62, *p* = 1.27 × 10^−27^). As shown in Table 1, the hazard ratios (HRs) for PRS values in the upper 2nd, 5th, 10th, and 20th percentiles consistently increased with higher PRS percentiles in both Model 1 and Model 2.

**Figure 2:**
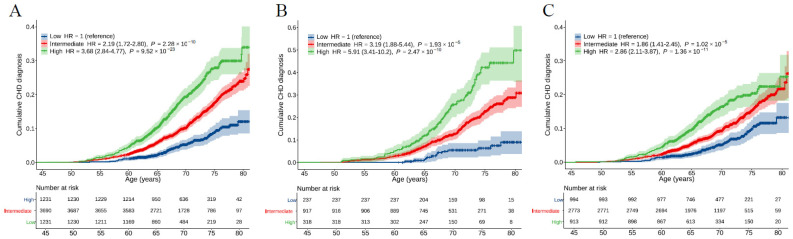
Cumulative incidence curves for CHD across polygenic risk categories. (**A**–**C**), Cumulative incidence curves were obtained from Kaplan–Meier estimates for CHD PRS in all participants (**A**), participants with hypertension (**B**), and participants without hypertension (**C**). The Cox proportional hazards model was used to estimate the HR (95% CI) and the cumulative risk of CHD adjusted for sex and the first ten principal components, with age as the time scale. Polygenic risk categories: low (bottom quintile), intermediate (2nd–4th quintile), or high (top quintile) risk according to quintiles of the CHD PRS. CHD, coronary heart disease; HR, hazard ratio.

We evaluated the association between CHD PRS and the lifetime (till 80 years of age) trajectories of CHD risk. The cumulative risk by age of 80 years for those in low, intermediate, and high genetic risk categories was 12.1%, 24.8%, and 33.9%, respectively (Figure 2A). In time-to-event analyses, the risk of CHD was higher in participants with intermediate (HR = 2.19, 95%CI: 1.72–2.80, *p* = 2.28 × 10^−10^) and high (HR = 3.68, 95%CI: 2.84–4.77, *p* = 9.52 × 10^−23^) genetic risk compared with those with low genetic risk. The Kaplan–Meier survival analyses, with follow-up time as the time scale, showed that the CHD PRS provided a significant gradient of CHD risk stratification (Figure S2A, log-rank test, *p* < 0.0001).

### 3.3. Interaction of Chronic Diseases with PRS for CHD Prediction

**Figure 3:**
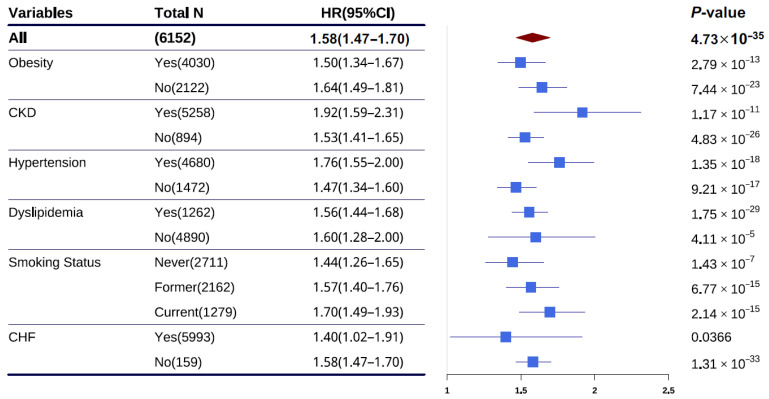
HR for CHD per 1-SD increase in PRS by subgroups. HR and 95% confidence interval were estimated using the Cox proportional hazards model, with follow-up time as the time scale, (adjusted for age, sex, the first 10 principal components) in different subgroups. In the right panel, blue squares and horizontal lines represent the HRs and their confidence intervals, respectively; regarding brown diamond, the center signifies the HR and the width represents the confidence interval. CHD, coronary heart disease; CI, confidence interval; HR, hazard ratio; SD, standard deviation; CKD, congestive kidney disease; CHF, coronary heart failure.

The association of the CHD PRS with CHD risk was investigated across strata of chronic diseases and smoking status. We found that the association of the CHD PRS with CHD risk was largely independent of chronic diseases and smoking status, as well as the ASCVD risk score (Table S4). Furthermore, we examined the association of the CHD PRS with CHD risk in different chronic disease populations. The HR was significantly higher in individuals with hypertension (HR = 1.76, 95%CI: 1.55–2.00, *p* = 1.35 × 10^−18^) compared with those without hypertension (HR = 1.47, 95%CI: 1.34–1.60, *p* = 9.21 × 10^−17^; *p* = 0.0144 for interaction, Figure 3 and Table S5). In contrast, HRs did not significantly differ in obesity (*p* = 0.263 for interaction), CKD (*p* = 0.0879 for interaction), dyslipidemia (*p* = 0.898 for interaction), CHF (*p* = 0.527 for interaction) or different smoking status (*p* = 0.364 for interaction) populations. Specifically, the individuals with obesity had lower HR (HR = 1.50, 95%CI: 1.34–1.67, *p* = 2.79 × 10^−13^) than those without obesity (HR = 1.64, 95%CI: 1.49–1.81, *p* = 7.44 × 10^−23^), the individuals with CKD had lower HR (HR = 1.92, 95%CI: 1.59–2.31, *p* = 1.17 × 10^−11^) than those without CKD (HR = 1.53, 95%CI: 1.41–1.65, *p* = 4.83 × 10^−26^), the individuals with dyslipidemia had lower HR (HR = 1.56, 95%CI: 1.44–1.68, *p* = 1.75 × 10^−29^) than those without dyslipidemia (HR = 1.60, 95%CI: 1.28–2.00, *p* = 4.11 × 10^−05^), the individuals with CHF have lower HR (HR = 1.40, 95%CI: 1.02–1.91, *p* = 3.66 × 10^−02^) than those without CHF (HR = 1.58, 95%CI: 1.47–1.70, *p* = 1.31 × 10^−33^). Similarly to the results in all individuals, the CHD PRS provided a significant gradient of CHD risk stratification in individuals with and without hypertension (Figure S2). Individuals with hypertension at high genetic risk exhibited a significantly lower probability of CHD event-free survival compared to those without hypertension. In time-event analyses, the risk of CHD for hypertensive individuals was elevated in those with intermediate (HR = 3.19, 95%CI: 1.88–5.44, *p* = 1.93 × 10^−5^) and high (HR = 5.91, 95%CI: 3.41–10.2, *p* = 2.47 × 10^−10^) genetic risk compared with those with low genetic risk (Figure 2B). Conversely, the risk of CHD for non-hypertensive individuals was attenuated in those with intermediate (HR = 1.86, 95%CI: 1.41–2.45, *p* = 1.02 × 10^−5^) and high (HR = 2.86, 95%CI: 2.11–3.87, *p* = 1.36 × 10^−11^) genetic risk compared to those with low genetic risk (Figure 2C). In addition, the cumulative risk of CHD by age of 80 years was significantly elevated in hypertensive individuals but attenuated in non-hypertensive individuals.

### 3.4. Addition of PRS to Clinical ASCVD Risk Score

We investigated whether adding the CHD PRS to the clinical ASCVD risk score improves predictive performance. By calculating the C-index of the Cox proportional hazards model, we evaluated the performance of models. We found that a model incorporating only PRS produced a C-index of 0.71 (95%CI: 0.69–0.73), whereas a model incorporating only clinical ASCVD risk score increased the model performance to 0.72 (95%CI: 0.70–0.74), and the PRS-enhanced model further improved performance to 0.74 (95%CI: 0.72–0.77, ΔC-index: 0.02, *p* = 0.004; Table S6). Furthermore, the PRS-enhanced model significantly improved the C-index in individuals with hypertension (*p* = 7.92 × 10^−04^), whereas no significant improvement was observed in non-hypertensive individuals (*p* = 0.192). When evaluating reclassification metrics in detail, we found there was significant reclassification improvement in three-category risk (<7.5%, 7.5–20%, >20%) assessment (NRI, 0.10, 95%CI: 0.04–0.16; continuous NRI, 0.47, 95%CI: 0.34–0.57, Table 2) across all participants. Furthermore, subgroup analysis revealed that the PRS-enhanced model performed better in hypertensive individuals (NRI, 0.13, 95%CI: 0.04–0.23; continuous NRI, 0.51, 95%CI: 0.35–0.67) compared with non-hypertensive individuals (NRI, 0.10, 95%CI: 0.05–0.16; continuous NRI, 0.37, 95%CI: 0.25–0.53, Table S7). Calibration analysis indicated that the model with both the clinical ASCVD risk score and PRS was well-calibrated in whole participants and different subgroups (Figure S3).

**Figure 4:**
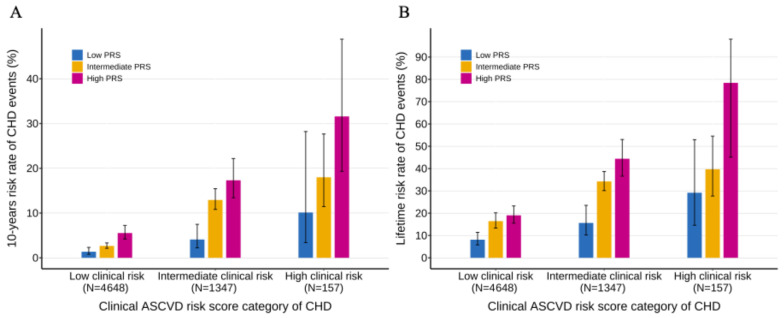
Ten-year and lifetime risk of CHD according to clinical ASCVD and polygenic risk categories. (**A**) Ten-year risk of CHD obtained from the clinical ASCVD risk score and CHD PRS model with follow-up time as the time scale. (**B**) Lifetime risk of CHD (till 80 years of age) obtained from the clinical ASCVD risk score and CHD PRS model, with age as the time scale. Participants were stratified into low (<7.5%), intermediate (7.5–20%), and high (>20%) ASCVD 10-year risk of CHD categories, and low (bottom quintile), intermediate (the second to fourth quintile), and high (top quintile) PRS risk categories. Within each clinical risk category, increased genetic risk conferred significantly increased risk of CHD (*p*-trend < 0.001 within each clinical risk categories). CHD, coronary heart disease; PRS, polygenic risk score; ASCVD, arteriosclerotic cardiovascular disease.

We further investigated the interaction of the PRS and the clinical ASCVD risk score on CHD risk. When the study population was stratified into ASCVD risk score categories, we observed that the PRS provided an additional significant gradient in the 10-year lifetime risk of CHD within each ASCVD risk score category (*p*-trend < 0.001) (Figure 4). When using both the ASCVD risk score and the PRS together, there was a nearly 23-fold and 10-fold increase in 10-year and lifetime risk from the lowest risk to the highest risk subgroups (1.36% 10-year risk and 8.15% lifetime risk in those with low clinical risk and low genetic risk; 31.58% 10-year risk and 78.43% lifetime risk in those with high clinical risk and high genetic risk). Furthermore, the ASCVD risk score provided a significant gradient of risk stratification across genetic risk categories. However, the estimate for the high-risk clinical group is characterized by a wide confidence interval, suggesting considerable uncertainty. This is most probably attributable to the subgroup’s small sample size (*n* = 157), which makes the estimate susceptible to sampling variability. Further study needs to be conducted. Similar analysis was conducted in the hypertensive population and the non-hypertensive population, and we observed similar patterns of the interaction between ASCVD risk score and PRS (Figure S4). We also observed that the 10-year and lifetime risk of CHD were elevated in individuals with hypertension but attenuated in individuals without hypertension.

## 4. Discussion

In this paper, we proposed a CHD PRS that has good performance in risk stratification of incident CHD risk, and enhanced the performance of the PCE when integrating this PRS into the clinical ASCVD risk score in the European ancestry population aged from 44 to 65. Meanwhile, we found that hypertension has an interaction with CHD PRS for predicting CHD risk. The time-to-event analyses demonstrated there was a substantial gradient of 10-year and lifetime CHD risk across polygenic risk categories. These results provide promising evidence that PRS can improve accuracy in identifying the risk of incident CHD for the European ancestry population, especially in the hypertensive population.

In recent decades, GWASs have demonstrated thousands of genetic loci associated with hundreds of phenotypes [33]. The PRSs derived from these GWASs can explain some phenotypic variance and have been used for disease prediction and risk stratification. Previous studies [8,19] have developed PRSs for CHD risk prediction, but they used old algorithms to calculate PRSs that may have unsatisfactory performance. Recently, there has been an increasing number of novel algorithms developed for improving the accuracy of PRSs in explaining phenotypic variance and risk prediction. This study used three advanced and recent algorithms to calculate PRSs and selected an optimal PRS for subsequent CHD risk prediction and population stratification. Our results demonstrate that the PRS calculated from the LDpred2 algorithm has the best performance, with a significant HR of 1.58 per 1-SD increment, AUC of 0.706, and C-index of 0.71. These values are higher than those reported in previous studies [18,34,35]. In our adjusted model, we found that those individuals in the highest PRS percentile had 10.4 times higher odds of having incident CHD than those in the lowest percentile. Our evaluation of the PRS risk stratification performance revealed that the individuals with high (HR = 3.66) and intermediate (HR = 2.19) genetic risk showed a significantly increasing CHD risk compared with those with low genetic risk, respectively. We observed that the HRs increased progressively with higher PRS percentiles (top 2nd, 5th, 10th, and 20th percentiles), demonstrating the excellent risk stratification performance of our CHD PRS. Our findings support the clinical utility of the proposed CHD PRS, showing enhanced accuracy in both CHD prediction and risk stratification. However, the predictive performance could potentially be further improved through more advanced algorithms (e.g., ensemble learning methods) and larger sample sizes.

Furthermore, our proposed CHD PRS was independent of conventional clinical risk factors (e.g., stroke, obesity), which illustrated the independent predictive value of the CHD PRS. Interestingly, in accordance with previous studies [36], we found that hypertension significantly modified the association between the PRS and CHD risk (*p* = 0.014). To our knowledge, there are few studies that investigate the interaction of hypertension with PRSs for predicting CHD risk. Further stratified analyses were conducted to evaluate the association between the PRS and CHD risk prediction across hypertension-specific subgroups. We observed that HR was significantly elevated (1.76 vs. 1.47, *p* = 0.014) in the population with hypertension compared to those without hypertension. Furthermore, among individuals with hypertension, those with high genetic risk had a higher incidence rate and HR than those with low genetic risk. These findings suggest that the PRS provides stronger CHD risk stratification in the hypertensive population.

The clinical utility of PRSs in CHD risk prediction and risk classification has been controversial when integrating the CHD PRS into a clinical risk score. Multiple studies [17,18,35,37] have reported improved predictive performance when adding the PRS to traditional clinical risk scores, whereas other investigations [19,20,34] found no significant enhancement in prediction accuracy. This discrepancy may reflect differences in study populations, PRS construction methods, or clinical risk score selection. We further investigated the reclassification performance of the addition of the PRS to the clinical risk score. Our PRS-enhanced model demonstrated excellent goodness-of-fit with a C-index of 0.74, which was significantly elevated compared with PCE (C-index of 0.72, *p* = 0.004). Adding the PRS to the clinical ASCVD risk score yielded a category NRI of 10% at three-category clinical ASCVD risk (<7.5%, 7.5–20% and >20%) and a continuous NRI of 47%. These findings demonstrate that the addition of the PRS improves CHD risk classification beyond the clinical ASCVD risk score. Similar trends were observed in the hypertensive population; however, the model performance declined slightly, with a reduction in the C-index from 0.74 to 0.71 and in NRI from 10% to 4%. This attenuation might be attributed to the limited sample size of hypertensive participants. Conversely, the C-index and NRI show no changes in the non-hypertensive population. Of note, the NRI would be 1.8% when assessing the reclassification in a two-category clinical ASCVD risk with a threshold of 7.5% in the ARIC cohort [18]. Though the improvement in discrimination is slight, in a primary prevention context for hypertensive population, the integration of the CHD PRS could potentially aid in refining risk stratification, particularly for those at intermediate risk. For such individuals, a high risk might promote more active management of other risk factors. Conversely, a low risk could reinforce the value of lifestyle interventions. Future studies are needed to determine whether CHD PRS-guided therapy leads to improved patient outcomes in real-world clinical practices. In our three-category risk strata, we observed a striking gradient of longitudinal risk across CHD PRS categories. Our PRS demonstrated a consistent capability to effectively stratify the trajectories of CHD genetic risk within each clinical ASCVD risk category. Notably, hypertensive individuals with high CHD PRS values exhibited an elevated incidence of CHD. These findings will be helpful for clinical practice.

The integration of CHD PRSs into routine CHD risk assessment necessitates careful consideration of its cost-effectiveness and ethical implications. From an economic perspective, the upfront costs of genotyping and implementation are non-negligible. However, several studies have reported that a prevention strategy guided by PRS could prove cost-effective in the long term by preventing expensive disease events in high-risk individuals [38,39]. Future health-economic modeling studies based on our findings are warranted to confirm this potential. Ethically, several challenges must be addressed. Firstly, the suboptimal performance of current PRSs in non-European populations raises serious concerns about health disparities. Since these PRSs are largely derived from European-ancestry GWASs, their clinical application could exacerbate existing health inequities [40]. Diversifying genetic studies is a moral and scientific imperative. Secondly, stringent policies are needed to protect genetic privacy and prevent misuse of this sensitive information.

Despite numerous studies assessing whether CHD PRSs improve CHD risk prediction beyond clinical risk scores, the results remain conflicting. Our study created several PRSs with powerful performance by using recent advanced algorithms and selected a best-performing PRS; the best-performing PRS outperformed previously published PRSs. Meanwhile, integrating our novel CHD PRS into the clinical ASCVD risk score offered substantial improvements in CHD risk prediction and stratification compared with the clinical ASCVD risk score alone. We observed an HR of 3.68 among those in the top 20% of the PRS values compared with the remaining 80% in the hypertensive population, supporting the evidence that the CHD PRS may serve as a risk-enhancing factor for CHD [41]. Furthermore, multiple studies have demonstrated the association of CHD PRS with disease susceptibility in demographic subgroups (e.g., male populations) [35,37], whereas its utility in chronic disease cohorts has not been systematically investigated. For instance, hypertension was believed to be associated with CHD, and a study reported that the risk of ASCVD in patients with resistant hypertension was significantly increased [42]. However, to the best of our knowledge, no previous study has investigated the interaction between the CHD PRS and hypertension in CHD risk prediction. We were the first to find this significant interaction. Our findings support the potential utility of PRSs in enhancing CHD risk prediction among patients with chronic diseases (e.g., hypertension), though further validation is warranted.

This work has some potential limitations. Firstly, this work is limited to the European ancestry population, and previous work has shown that PRSs developed and tested based on different populations may experience performance degradation [43]. Therefore, the predictive performance of our CHD PRS is attenuated when applied to non-European populations, due to differences in genetic architecture. Furthermore, the insufficient GWAS data from non-European ancestry groups led to limited prediction performance. Fortunately, numerous cross-ancestry PRS algorithms have been developed, and GWAS summary data for diverse ancestry have been released and are publicly available. In the future, we could develop many transferable cross-ancestry PRSs for non-European ancestral populations to reduce the prevailing inequities in genetic and health research. Secondly, the ARIC cohort is one of the cohorts in CARDIoGRAMplusC4D, which might lead to an overestimation of the performance of the PRS. However, we note that ARIC constituted approximately 0.9% of the total discovery sample size. The genetic variants’ weights were predominantly determined by the vast majority of non-ARIC samples, which likely attenuated the severity of overfitting. Therefore, the potential inflation of performance metrics is slight. Thirdly, the limited sample size in our study may have led to an overestimation of both CHD risk and incidence rates. For instance, while the general population prevalence of CHD is 7.2%, our cohort exhibited a higher prevalence of 12.5%. Fourthly, the performance of our PRS-enhanced risk model may differ across varying ASCVD 10-year CHD risk thresholds; therefore, further study needs to be conducted. In addition, a new model called SCORE2 [5] has more powerful 10-year risk prediction ability and more accurate population stratification ability than PCE, it can be used for further analysis in the future. Fifthly, as this study is restricted to participants aged 44–65 years, the findings may not be generalizable to younger (<44 years) or older (>65 years) populations. Genetic testing at an earlier age may further improve risk prediction accuracy, but this hypothesis requires validation in future studies. Finally, the present study focused on adding PRS to clinical risk factors and ignored the impacts of socio-demographic, family history, lifestyle, and other environmental variables. Previous works [8,44,45] have shown that those variables have significant impacts on disease prediction.

## 5. Conclusions

We proposed a CHD PRS that has good performance in risk stratification of incident CHD beyond the clinical ASCVD risk score. Furthermore, we observed there were interactions between hypertension and the PRS for predicting CHD risk. PRSs may provide valuable CHD risk stratification guidance to identify hypertensive patients who should initiate intensive lifestyle changes or be given drug treatments.

## Figures and Tables

**Table 1:** Hazard ratios of CHD events for the PRSs (per 1-SD increase) and selected PRS strata.

PRS	Model 1		Model 2	
	HR (95%CI)	*p*-Value	HR (95%CI)	*p*-Value
Continuous per SD increment	1.58 (1.47, 1.70)	4.73 × 10^−35^	1.51 (1.40, 1.62)	1.27 × 10^−27^
Top 20%	2.53 (2.00, 3.21)	1.58 × 10^−14^	2.37 (1.87, 3.01)	1.26 × 10^−12^
Top 10%	2.75 (1.95, 3.88)	8.80 × 10^−09^	2.49 (1.76, 3.51)	2.38 × 10^−07^
Top 5%	3.32 (1.96, 5.64)	8.88 × 10^−06^	3.07 (1.80, 5.22)	3.49 × 10^−05^
Top 2%	6.14 (1.97, 19.1)	0.00172	5.86 (1.88, 18.2)	0.00223

HR and 95% CI derived from Cox proportional hazards models; model 1 adjusted for age, sex, and first ten principal components; model 2 adjusted for age, sex, and first ten principal components, diabetes, LDL-C, HDL-C, SBP, DBP, hypertension, antihypertension, and smoking status. HR, hazards ratios; CI, confidence interval; PRS, polygenic risk score; CHD, coronary heart disease; HDL-C: high-density lipoprotein cholesterol; LDL-C: low-density lipoprotein cholesterol; SBP, systolic blood pressure; DBP, diastolic blood pressure.

**Table 2:** Net reclassification improvement after adding CHD PRS to PCE ASCVD risk score.

PCE Model	PRS-Enhanced Model			
	<7.5%	7.5–20%	>20%	Total
**CHD**				
<7.5%	126	45	1	172
7.5–20%	20	102	17	139
>20%	0	4	18	22
Total	146	151	36	333
**Non-CHD**				
<7.5%	4401	337	1	4739
7.5–20%	302	605	67	974
>20%	1	17	54	72
Total	4704	959	122	5785
**Net reclassified improvement**				
NRI for CHD (95%CI)				0.12 (0.06, 0.17)
NRI for Non-CHD (95%CI)				−0.01 (−0.02, −0.01)
NRI (95%CI)				0.10 (0.04, 0.16)
Continuous NRI for CHD (95%CI)				0.26 (0.14, 0.37)
Continuous NRI for Non-CHD (95%CI)				0.21 (0.18, 0.24)
Continuous NRI (95%CI)				0.47 (0.34, 0.57)

In the upper part, columns and rows refer to categories of 10-year predicted risk, and the numbers represent the counts of individuals assigned to the indicated risk category. The standard CHD model adjusted for sex, age, the first ten principal components, and the low (<7.5%), intermediate (7.5–20%) and high (>20%) risk categories are based on the ASCVD risk score. NRI, net reclassification improvement; CHD, coronary heart disease; CI, confidence interval; PRS, polygenic risk score; NRI, net reclassified improvement; ASCVD, arteriosclerotic cardiovascular disease.

## Data Availability

GWAS summary statistics data is available at https://cvd.hugeamp.org/ (accessed on 6 March 2025). The ARIC phenotypic and genetic data are available on the database of Genotypes and Phenotypes (dbGaP; study accession: phs0000280.v8.p2). The R package bigsnpr (version 4.3.1) used to conduct P + T and LDpred2 algorithms is available at https://github.com/privefl/bigsnpr (accessed on 7 March 2025). The software PRS-cs is available at https://github.com/getian107/PRScs (accessed on 11 March 2025).

## Supplementary Materials

The following supporting information can be downloaded at https://www.mdpi.com/article/10.3390/jcdd12120454/s1.
