# Supplementary material for: Enhanced Risk Prediction for Coronary Heart Disease by Leveraging Polygenic Risk Score and Clinical Risk Score in European Hypertensive Adults
Source: J Cardiovasc Dev Dis. 2025 Nov 24;12(12):454. doi: 10.3390/jcdd12120454 (PMC12734198; doi:10.3390/jcdd12120454)
Supplement: Supplementary file 1 [file jcdd-12-00454-s001.zip › jcdd-3941166-supplementary.pdf]

Supplemental materials

Calculation of polygenic risk score

The Polygenic risk score (PRS) of an individual *i* is the weighted sum of risk alleles based on the GWAS summary data using the following formula

$$PRS_i = \sum_{j=1}^{M} \beta_j \cdot G_{ij},$$

where β_j is the effect size of the causal allele for SNP j and G_{ij} coded as 0, 1 and 2 is the number of the causal allele for SNP j. We looked at three methods for PRS calculation:

P+T selects SNPs based on clumping and thresholding applied to a series of combinations of *P*-values (10^{-6}, 10^{-5}, 10^{-4}, 10^{-3}, 0.005, 0.01, 0.05, 0.1, 0.5 and 1) and SNP linkage disequilibrium correlation r^2 (0.1, 0.4 and 0.8), the remained SNPs are used for calculating PRS. We then selected the optimal r^2 and *P*-value threshold via parameter tuning on a validation dataset.

LDpred2 is a Bayesian approach that accounts for linkage disequilibrium by jointly modeling SNP effect sizes, and offers improved computational efficiency and robustness compared to its predecessor LDpred. Given a linear model $\boldsymbol{y} = \boldsymbol{G\beta} + \boldsymbol{\varepsilon}$, LDpred2 places an independent spike-and-slab prior on each effect size β_j, each SNP has a non-zero causal effect $\beta_j \sim N(0, h_g^2/\pi M)$ with probability π and has no contribution to phenotypic variance ($\beta_j = 0$) with probability $(1-\pi)$, where h_g^2 is the heritability explained by genome-wide SNPs for phenotype $\boldsymbol{y}$; π is the proportion of casual SNPs. In this study, we developed a 'grid' version, which estimates the weight of SNP under a range of combination of sparsity π (0.001, 0.003, 0.01, 0.03, 0.1, 0.3 and 1) and total heritability h_g^2 which is set to the heritability estimated by linkage disequilibrium score regression (LDSC) multiplied by 0.7, 1, or 1.4. The best parameters of π and h_g^2 were selected via parameter tuning on a validation dataset. Furthermore, we developed an 'auto' version, where LDpred2 automatically estimates π and h_g^2.

PRS-cs applies a Bayesian shrinkage parameter on effect size estimates for each SNP: $\beta_j \sim N\left(0, \sigma^2 \phi \psi_j / N\right), \psi_j \sim G\left(a, \delta_j\right), \delta_j \sim G(b, 1)$, where σ^2 is the residual variance; ϕ is a global shrinkage parameter that is shared across all effect sizes; ψ_j is a local and SNP-specific parameter; $G(\alpha, \beta)$ denotes the gamma distribution with shape parameter α and scale parameter β. In our analyses, we ran PRS-cs with the default candidate value for the tuning global shrinkage parameters ϕ. An independent validation dataset was used to select optimal parameter. Furthermore, the 'auto' version of PRS-cs which can automatically estimate ϕ, was developed.

Calculation of R^2 on the liability scale

Due to the status of coronary heart disease (CHD) is a binary variable, we calculated the proportion of variation in CHD status explained by the PRS on the liability scale using following equation:

$$R_{liability}^2 = R_{observed}^2 \cdot \frac{z_K^2}{K(1-K)} \cdot \frac{Q(1-Q)}{z_Q^2},$$

where $R_{observed}^2$ is the pseudo R^2 of logistic regression, *K* and *Q* are the CHD incidence rate in general population and in our study cohort, respectively; z_K^2 and z_Q^2 are the probability density of the standard normal distribution at the thresholds *K* and *Q*, respectively

Table S1 Baseline characteristics of study cohort by clinical risk score.

Baseline characteristics	Overall N = 6,152 [a]	High N = 157 [a]	Intermediate N = 1,347 [a]	Low N = 4,648 [a]	*P*-value [b]
Age, years	54.02 (5.63)	60.50 (3.57)	57.73 (4.91)	52.73 (5.24)	3.29×10^{-220}
Gender, N(%)					5.80×10^{-260}
Female	3,400.00 (55.27%)	15.00 (9.55%)	238.00 (17.67%)	3,147.00 (67.71%)	
Male	2,752.00 (44.73%)	142.00 (90.45%)	1,109.00 (82.33%)	1,501.00 (32.29%)	
HDL-C, mg/dL	51.94 (16.61)	39.37 (10.23)	41.83 (11.21)	55.30 (16.71)	5.94×10^{-204}
LDL-C mg/dL	136.17 (36.95)	156.65 (40.65)	150.31 (34.60)	131.38 (36.20)	1.01×10^{-74}
Systolic blood pressure, mmHg	117.70 (16.30)	138.41 (18.15)	126.69 (16.40)	114.39 (14.62)	7.10×10^{-168}
Diastolic blood pressure, mmHg	71.65 (9.77)	78.20 (10.60)	74.59 (10.10)	70.57 (9.37)	6.46×10^{-52}
Diabetes, N(%)	394 (6.40%)	103 (65.61%)	168 (12.47%)	123 (2.65%)	8.39×10^{-242}
Smoking Status, N(%)					1.98×10^{-93}
Current	1,279 (20.79%)	67 (42.68%)	497 (36.90%)	715 (15.38%)	
Former	2,162 (35.14%)	57 (36.31%)	507 (37.64%)	1,598 (34.38%)	
Never	2,711 (44.07%)	33 (21.02%)	343 (25.46%)	2,335 (50.24%)	
Hypertension, N(%)	1,472 (23.93%)	106 (67.52%)	536 (39.79%)	830 (17.86%)	3.44×10^{-97}
Antihypertensives, N(%)	1,056 (17.17%)	84 (53.50%)	371 (27.54%)	601 (12.93%)	2.85×10^{-67}
eGFR	71.35 (11.11)	69.38 (12.94)	71.06 (11.56)	71.51 (10.90)	0.021
Obesity, N(%)	2,122 (34.49%)	89 (56.69%)	581 (43.13%)	1,452 (31.24%)	1.50×10^{-22}
Dyslipidemia, N(%)	4,890 (79.49%)	150 (95.54%)	1,237 (91.83%)	3,503 (75.37%)	5.65×10^{-44}
CKD, N(%)	894 (15%)	37 (24%)	217 (16%)	640 (14%)	5.02×10^{-04}
CHF, N(%)	159 (2.58%)	8 (5.10%)	43 (3.19%)	108 (2.32%)	0.028
Stroke, N(%)	84 (1.37%)	2 (1.27%)	17 (1.26%)	65 (1.40%)	0.94
CHD event, N(%)					1.09×10^{-81}
CHD events	767 (12.47%)	57 (36.31%)	341 (25.32%)	369 (7.94%)	
Non-CHD events	5,385 (87.53%)	100 (63.69%)	1,006 (74.68%)	4,279 (92.06%)	
Follow up time, years	15.47 (2.81)	13.11 (4.52)	14.38 (3.89)	15.87 (2.17)	1.32×10^{-41}

Abbreviations: CHD, coronary heart disease; HDL-C: high-density lipoprotein cholesterol; LDL-C: low-density lipoprotein cholesterol; eGFR, estimated Glomerular Filtration Rate; CKD, chronic kidney disease; CHF, congestive heart failure.

[a] Mean (SD); n (%)

[b] Kruskal-Wallis rank sum test; Pearson's Chi-squared test; Fisher's exact test

Table S2 Prediction accuracy of the six PRSs.

PRS	Observed R^2	Liability R^2	AUC (95%CI)	OR (95%CI)
Khera	0.30%	0.40%	0.679(0.644, 0.718)	1.13(1.04, 1.22)
PRS-CS-auto	0.36%	0.49%	0.680(0.652, 0.725)	1.14(1.06, 1.24)
PRS-CS	0.21%	0.29%	0.679(0.649, 0.725)	1.11(1.02, 1.20)
P+T	0.45%	0.61%	0.681(0.659, 0.713)	1.16(1.07, 1.26)
LDpred-auto	3.54%	4.76%	0.710(0.682, 0.761)	1.54(1.42, 1.67)
LDpred	4.69%	6.30%	0.719(0.680, 0.764)	1.65(1.52, 1.79)

Khera PRS means we used the SNP weights obtained from his publication. Others PRSs correspond to their SNP weights derive algorithms. Observed R^2 was derived from logistic regression, and liability R^2 was calculated using observed R^2 multiply an adjusted factor calculated through both prevalence in real population and in study cohort. AUC and OR were calculated using logistic regression (adjusted for age, sex, the first 10 principal components), respectively. 95%CI was obtained by 10-fold cross validation with 100 replications. AUC, area under curve; OR, odds ratio.

Table S3 Hazard ratios of incident CHD for the PRS (per 1-SD increase) after adjustment for chronic disease and ASCVD risk score.

Characteristic	HR	95% CI	*P*-value
CHD PRS	1.58	(1.47, 1.70)	4.73×10^{-35}
CHD PRS+ Obesity	1.56	(1.45, 1.68)	1.07×10^{-33}
CHD PRS+ CKD	1.58	(1.47, 1.70)	4.22×10^{-35}
CHD PRS+ Hypertension	1.56	(1.45, 1.68)	4.43×10^{-33}
CHD PRS+ Dyslipidemia	1.56	(1.45, 1.68)	5.88×10^{-33}
CHD PRS+CHF	1.57	(1.46, 1.69)	1.39×10^{-34}
CHD PRS+ Smoking status	1.57	(1.46, 1.69)	2.03×10^{-34}
CHD PRS+ Stroke	1.58	(1.47, 1.70)	4.89×10^{-35}
CHD PRS+7 clinical risk factors	1.53	(1.42, 1.64)	3.93×10^{-30}
CHD PRS+ASCVD risk score	1.55	(1.45, 1.67)	7.35×10^{-33}

Dyslipidemia was defined as total cholesterol (TC) ≥240 mg/dL and/or low-density lipoprotein cholesterol (LDL-C) ≥160 mg/dL and/or triglycerides (TG) ≥200 mg/dL and/or high-density lipoprotein cholesterol (HDL-C) <40 mg/dL and/or the use of lipid-lowering medication within the past 2 weeks. For hypertension, the definition was systolic BP ≥140 mmHg and/or diastolic BP ≥90 mmHg and/or use of antihypertensive medications within the past 2 weeks. Diabetes was defined as fasting glucose levels ≥126 mg/dL and/or use of insulin and/or oral hypoglycemic agents and/or diagnosed medical history of diabetes. BMI was calculated as weight in kilograms divided by height in squared meters. Smoking status referred to self-reported status of cigarette smoking. HR and 95% confidence interval were estimated using Cox proportional hazards model with follow-up time as time scale (adjusted for age, sex, the first 10 principal components). CHD, coronary heart disease; CI, confidence interval; HR, hazard ratio; PRS, polygenic risk score; SD, standard deviation.

Table S4 Hazard ratios of CHD for the PRS (per 1-SD increase) in different subgroup and interaction between CHD PRS and chronic diseases.

Characteristic	Overall N = 6,152	HR	95% CI	*P*-value	Interaction *P*-value
**Obesity**					0.263
Yes	2,122	1.50	(1.34, 1.67)	2.79×10^{-13}	
No	4,030	1.64	(1.49, 1.81)	7.44×10^{-23}	
**CKD**					0.0879
Yes	894	1.92	(1.59, 2.31)	1.17×10^{-11}	
No	5,258	1.53	(1.41, 1.65)	4.83×10-^{26}	
**Hypertension**					0.0144
Yes	1,472	1.76	(1.55, 2.00)	1.35×10^{-18}	
No	4,680	1.47	(1.34, 1.60)	9.21×10^{-17}	
**Dyslipidemia**					0.898
Yes	4,890	1.56	(1.44, 1.68)	1.75×10^{-29}	
No	1,262	1.60	(1.28, 2.00)	4.11×10^{-05}	
**Smoking status**					0.364
Current	2,711	1.44	(1.26, 1.65)	1.43×10^{-07}	
Former	2,162	1.57	(1.40, 1.76)	6.77×10^{-15}	
Never	1,279	1.70	(1.49, 1.93)	2.14×10^{-15}	
**Stroke**					0.354
Yes	159	3.36	(1.22, 9.27)	1.91×10^{-02}	
No	5,993	1.57	(1.46, 1.69)	9.69×10^{-34}	
**CHF**					0.527
Yes	159	1.40	(1.02, 1.91)	3.66×10^{-02}	
No	5,993	1.58	(1.47, 1.70)	1.31×10^{-33}	

HR and 95% confidence interval were estimated using Cox proportional hazards model with follow-up time as time scale (adjusted for age, sex, the first 10 principal components) in different subgroups. Interaction *P*-value was calculated using Cox proportional hazards with follow-up time as time scale (adjusted for age, sex, the first 10 principal components and interaction term). CHD, coronary heart disease; CI, confidence interval; HR, hazard ratio; PRS, polygenic risk score; SD, standard deviation; CKD, congestive kidney disease; CHF, coronary heart failure.

Table S5 C-index and NRI for CHD PRS by hypertension status and ASCVD risk score.

Clinical ASCVD risk score	ALL		Hypertension		Non-hypertension	
	C-index (95%)	*P*-value [a]	C-index (95%)	*P*-value [a]	C-index (95%)	*P*-value [a]
ALL	0.71(0.69, 0.73) [b]<br>0.72(0.70, 0.74) [c]<br>0.74(0.72, 0.77) [d]	0.004	0.69(0.66, 0.72) [b]<br>0.66(0.62, 0.70) [c]<br>0.71(0.68, 0.75) [d]	7.92×10^{-04}	0.71(0.69, 0.73) [b]<br>0.72(0.70, 0.75) [c]<br>0.74(0.72, 0.76) [d]	0.192
Low	0.69(0.66, 0.72) [b]<br>0.69(0.66, 0.72) [c]<br>0.72(0.69, 0.74) [d]	0.005	0.68(0.59, 0.77) [b]<br>0.56(0.49, 0.62) [c]<br>0.68(0.6, 0.75) [d]	1.14×10^{-04}	0.69(0.66, 0.72) [b]<br>0.70(0.67, 0.74) [c]<br>0.72(0.69, 0.75) [d]	0.056
Intermediate	0.61(0.59, 0.62) [b]<br>0.57(0.55, 0.59) [c]<br>0.63(0.62, 0.65) [d]	1.25×10^{-04}	0.60(0.52, 0.67) [b]<br>0.52(0.47, 0.56) [c]<br>0.61(0.54, 0.68) [d]	3.09×10^{-04}	0.60(0.54, 0.66) [b]<br>0.58(0.53, 0.64) [c]<br>0.64(0.58, 0.70) [d]	2.57×10^{-05}
High	0.69(0.66, 0.72) [b]<br>0.69(0.66, 0.72) [c]<br>0.72(0.69, 0.74) [d]	0.143	0.68(0.59, 0.77) [b]<br>0.56(0.49, 0.62) [c]<br>0.68(0.60, 0.75) [d]	3.27×10^{-13}	0.69(0.66, 0.72) [b]<br>0.70(0.67, 0.74) [c]<br>0.72(0.69, 0.75) [d]	NA [e]

a, *P*-value was calculated for estimating the difference between PCE model (includes ASCVD risk score only) and PRS-enhanced model (includes PRS and ASCVD risk score); b, C-index for Cox model with PRS only; d, C-index for Cox model with ASCVD risk score only; d, C-index for Cox model with ASCVD risk score and PRS. e, the *P*-value of NA was caused by the limited sample. All models were constructed using Cox proportional hazards model with follow-up time as time scale (adjusted for age, sex, the first 10 principal components). 95%CI for C-index was obtained using 10-fold cross validation, and 95%CI for NRI was obtained using bootstrap algorithm. C-index, concordance index; CI, confidence interval; PRS, polygenic risk score; ASCVD, arteriosclerotic cardiovascular disease.

Table S6 Net reclassification improvement after adding CHD PRS to clinical ASCVD risk score in subgroups based on hypertension status grouping. a) Participants with hypertension and (b) participants without hypertension with low, intermediate and high clinical ASCVD risk, comparing a model that includes ASCVD risk score alone and one that includes ASCVD risk score + PRS, both models adjust for age, sex and the first 10 principal components

(a) Analysis in participants with hypertension with low, intermediate and high risk by clinical ASCVD risk score.

**PCE model**	**PRS-enhanced model**			
	**<7.5%**	**7.5%-20%**	**>20%**	**Total**
**CHD**				
<7.5%	20	13	0	33
7.5%-20%	12	52	13	77
>20%	0	2	10	12
Total	32	67	23	122
**Non-CHD**				
<7.5%	681	106	1	788
7.5%-20%	181	291	43	515
>20%	1	14	20	35
Total	863	411	64	1,338
**Net reclassified improvement**				
NRI for CHD (95% CI)				0.04(0.01, 0.06)
NRI for Non-CHD (95% CI)				0.10(0.01, 0.19)
NRI (95% CI)				0.13(0.04, 0.23)
Continuous NRI for CHD (95% CI)				0.26(0.09, 0.42)
Continuous NRI for Non-CHD (95% CI)				0.25(0.20, 0.30)
Continuous NRI (95% CI)				0.51(0.35, 0.67)

(b) Analysis in participants without hypertension with low, intermediate and high risk by clinical ASCVD risk score.

PCE model	PRS-enhanced model			
	**<7.5%**	**7.5%-20%**	**>20%**	**Total**
**CHD**				
<7.5%	97	26	0	123
7.5%-20%	10	58	8	76
>20%	0	1	11	12
Total	107	85	19	211
**Non-CHD**				
<7.5%	3,638	199	0	3,837
7.5%-20%	167	375	21	563
>20%	0	10	37	47
Total	3,805	584	58	4,447
**Net reclassified improvement**				
NRI for CHD (95%CI)				0.11(0.06, 0.18)
NRI for Non-CHD (95% CI)				-0.01(-0.02, -0.002)
NRI (95% CI)				0.10(0.05, 0.16)
Continuous NRI for CHD (95% CI)				0.21(0.11, 0.37)
Continuous NRI for Non-CHD (95% CI)				0.16(0.13, 0.18)
Continuous NRI (95% CI)				0.37(0.25, 0.53)

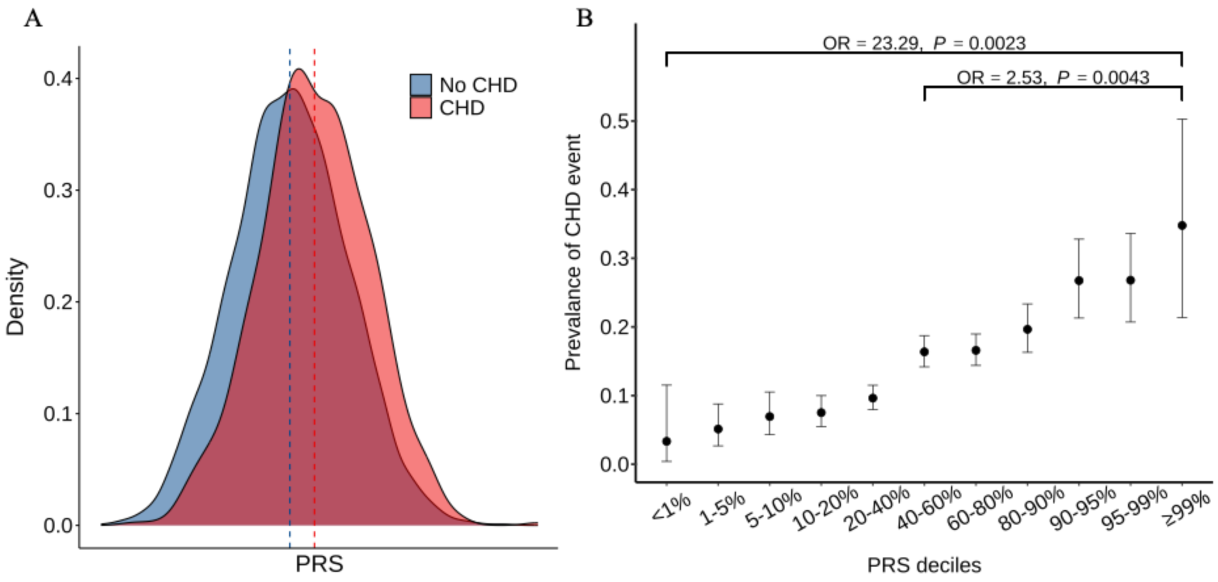

Figure S1 Genome-wide PRS and the risk of CHD. A, Density plots of PRR in CHD cases and non-cases, vertical lines represent the mean value of PRS in CHD cases and non-cases. **B,** CHD prevalence and risk in ARIC study cohort demonstrating significantly higher CHD prevalence in individuals with the highest PGS (top centile, n=62), compared with the median (n=1230) and lowest groups (bottom centile, n=62). Effect estimates generated using logistic regression adjusting for age, sex and first 10 principal components, with unadjusted two-sided *P* value. Data are presented as effect estimates with 95% CI.

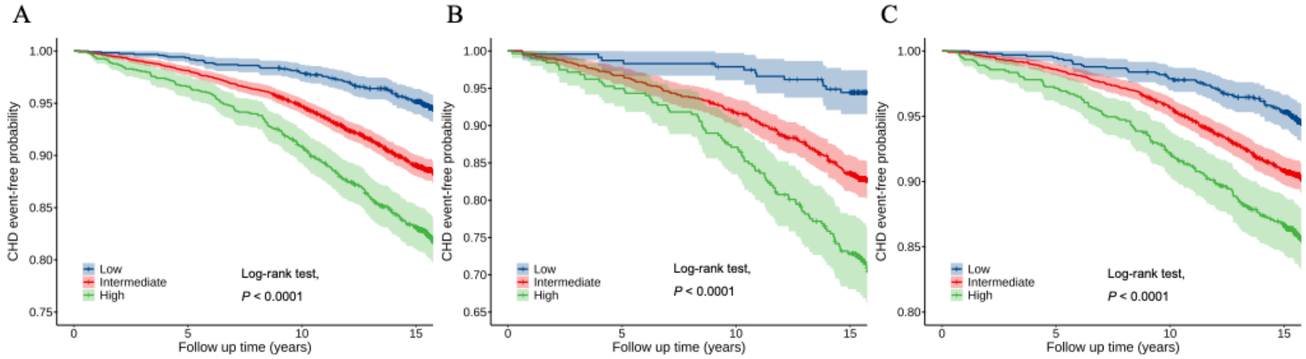

Figure S2 Event-free probability for CHD according to PRS. A-C, Event-free probability curves for CHD were obtained from Kaplan-Meier estimates for CHD PRS stratified into low (bottom quintile), intermediate (second–fourth quintile) and high (top quintile) polygenic risk in all participants (A) and separately in participants with hypertension (B) and without hypertension (C). Only individuals without previous event (before recruitment) were included for a median follow-up of 15.47 years (Log-rank test, P values <0.0001 for A, B, and C).

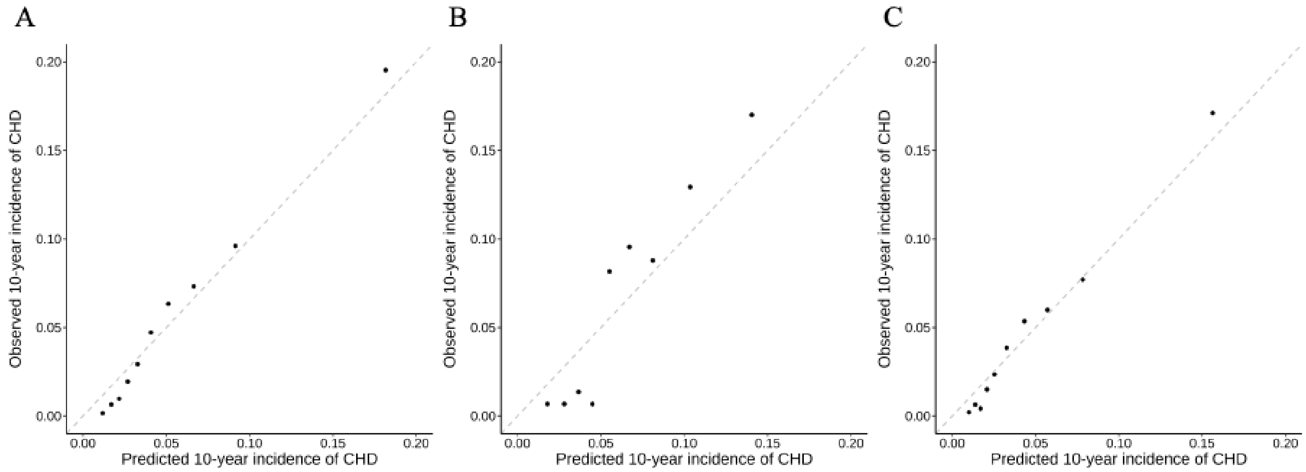

Figure S3 Calibration plot for the PRS-enhanced model in subgroups. A, Calibration plot for all participants. **B,** Calibration plot for participants with hypertension. C, Calibration plot for participants without hypertension. Recalibration was undertaken by estimating the baseline survival function and fitting the predicted hazard ratios as a covariate in a Cox regression model with follow-up time as the underlying time-scale. Visual inspection of the calibration curve demonstrates that on average the predicted cohort risk in the PRS-enhanced model is similar to the observed risk. PRS, polygenic risk score.

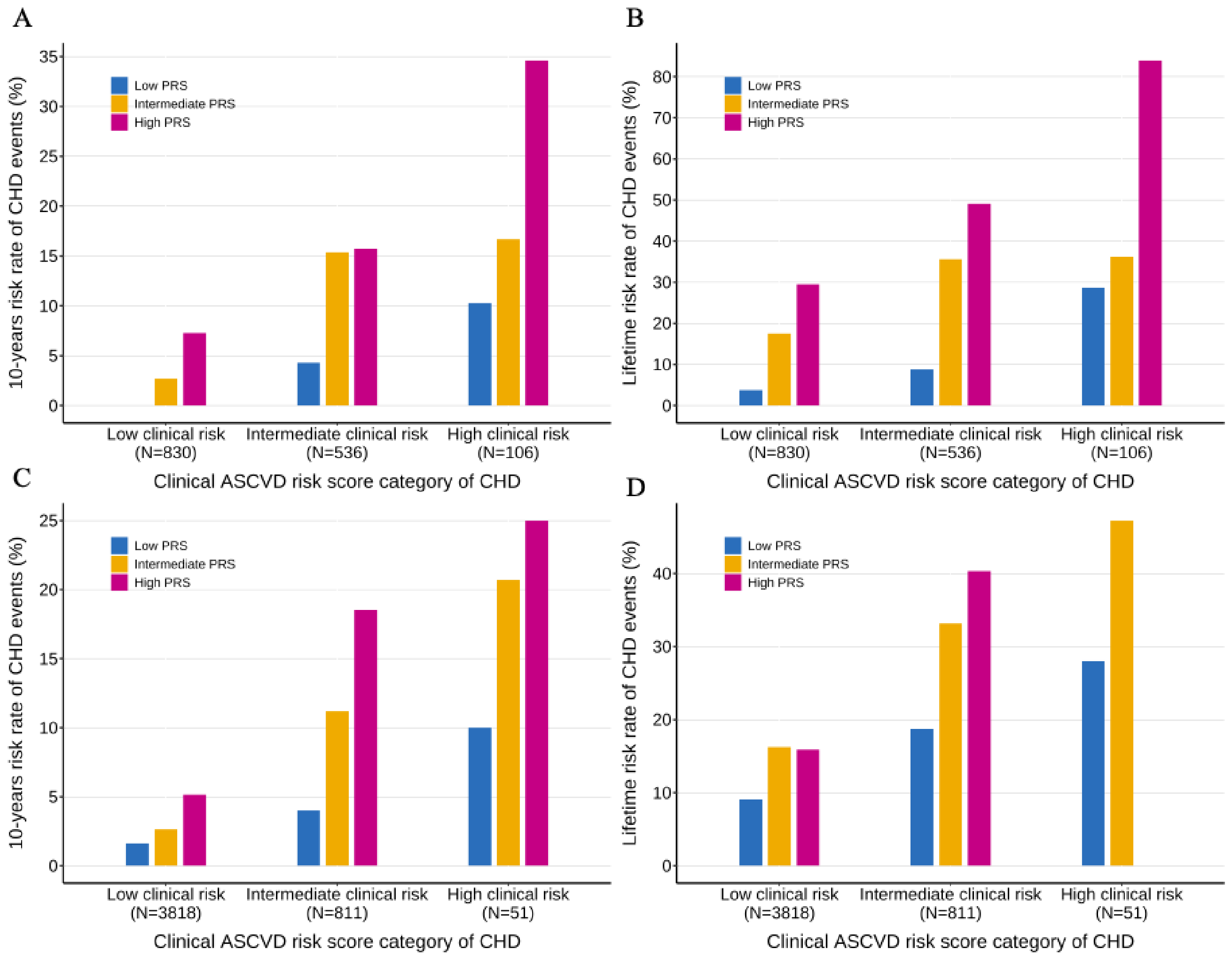

Figure S4 Ten-year and lifetime risk of CHD according to clinical ASCVD and polygenic risk categories in subgroups based on hypertension status grouping. A, C, Ten-year risk of CHD obtained from the clinical risk and CHD PRS model with follow-up time as the time scale in individuals with hypertension. **B, D,** Lifetime risk of CHD (till 80 years of age) obtained from the clinical risk and CHD PRS model with age as the time scale in individuals without hypertension. Participants were stratified into low (<7.5%), intermediate (7.5%-20%) and high (>20%) ASCVD 10-year risk of CHD categories, and low (bottom quintile), intermediate (the second to fourth quintile) and high (top quintile) PRS risk categories.
